# Information and Communication Technologies for Chronic Disease Self-Management in Adults Aged 65 Years and Older: Scoping Review

**DOI:** 10.2196/60542

**Published:** 2026-03-19

**Authors:** Paul Murdock, Yiyi Wu, Charles R Senteio

**Affiliations:** 1 Cleveland Clinic, 9500 Euclid Ave, Cleveland, OH, 44195, United States, 1 614-230-8109; 2 Rutgers University School of Communication and Information, New Brunswick, NJ, United States

**Keywords:** health technology, self-care, self-management, chronic disease, efficacy, technology, older adult, gerontology, chronic, chronic diseases, systematic review, behavioral health, health equity, Preferred Reporting Items for Systematic reviews and Meta-Analyses, PRISMA, medication behavior, dietary behavior, physical activity, physical rehabilitation, digital health

## Abstract

**Background:**

The increasing number of older adults living with chronic conditions has led to rapid growth in information and communication technologies (ICTs) designed to support chronic disease self-management. Although many technologies target behaviors such as medication adherence, physical activity, dietary management, and follow-up care, the breadth, characteristics, and design considerations of these tools for adults aged 65 years and older have not been comprehensively reported.

**Objective:**

This scoping review aims to systematically map the existing literature describing ICTs developed to support chronic disease self-management among adults aged 65 years and older. Specifically, the review seeks to (1) identify the types of ICTs available; (2) characterize the self-management behaviors they target; and (3) examine the extent of older adults’ involvement in the design, adaptation, or evaluation of these technologies.

**Methods:**

This review followed the PRISMA-ScR (Preferred Reporting Items for Systematic Reviews and Meta-Analyses extension for Scoping Reviews) guidelines. Seven databases (PubMed, CINAHL, Web of Science, Cochrane Library, Compendex, IEEE Xplore, and Computers & Applied Sciences Complete) were searched, with all searches completed on December 15, 2024. Inclusion criteria were peer-reviewed studies published in English between 2007 and 2025 that (1) included adults aged ≥65 years; (2) addressed one or more chronic diseases; and (3) evaluated, described, or tested an ICT intended to support at least 1 chronic disease self-management behavior. Two reviewers independently screened all titles and abstracts and full texts; disagreements were resolved by a third reviewer. Data were charted using a standardized extraction template and synthesized narratively by technology type and self-management domain.

**Results:**

Nineteen studies met the inclusion criteria. Technologies were grouped into 4 broad categories: mobile apps, online platforms, wearable or sensor-based tools, and smart home or device-integrated systems. Physical activity and medication management were the most targeted self-management behaviors, whereas follow-up appointment adherence and dietary behaviors were less frequently addressed. Only a small number of studies explicitly involved older adults in the design or development process, and such involvement was often limited to usability testing rather than participatory co-design.

**Conclusions:**

The current evidence base is fragmented, with substantial variability in technology types, targeted behaviors, and reported outcomes. Significant gaps remain regarding the participatory design of ICTs with older adults and the development of technologies that address multiple self-management needs simultaneously. Future ICT development should intentionally incorporate older adults and caregivers throughout the design cycle and expand beyond single-behavior interventions to reflect the multimorbidity common in this population.

## Introduction

Most population projections estimate that by 2030, 1 in 6 people worldwide will be aged 60 years or older [1], compared with 1 in 10 at present [2]. The older adult population is expected to grow and eventually double by 2050, reaching 2.1 billion persons worldwide [1]. According to the US Centers for Disease Control and Prevention, 6 in 10 adults in the United States are living with 1 or more of the “big five” chronic conditions: diabetes mellitus, cardiovascular disease, chronic respiratory disease, cancer, and stroke [3]. Older adults are at increased risk of having chronic conditions; two-thirds of Medicare beneficiaries have 2 or more chronic conditions [3]. This shifting demographic and the prevalence of chronic conditions result in an expanding number of older adults living with chronic conditions.

For individuals living with chronic diseases, effective self-management, wherein patients take an active role in their own care, is essential for improving physical health, emotional well-being, and overall quality of life [4]. Self-management typically involves patients consistently engaging in healthy lifestyle behaviors, including maintaining a balanced diet, participating in regular physical activity, adhering to prescribed medications, and attending follow-up medical appointments [5].

Rapid advancements in technology (ie, smartphones and applications), connectivity (ie, mobile broadband availability), and commercial potential have resulted in an explosion of numerous information and communications technology (ICT) tools designed to support chronic disease self-management behaviors [6-8]. For older adults in particular, technologies designed to support health care and chronic disease management are classified into four general areas, which are based upon location of use and platform: (1) mobile-based apps, (2) smart home–based technologies, (3) online-based technologies, and (4) personalized application-based technologies [9]. Mobile-based apps, typically accessed via smartphones and tablets, are widely used to support remote medical services and personalized care. Among these, mobile health and telehealth platforms are the most prevalent. Smart home technologies, which incorporate ICT-enabled tools within the home, such as digital reminders for medical appointments, are increasingly recognized for their role in chronic disease management. Online-based technologies mainly refer to web-based services, including access to podcasts, disease-specific forums, health care providers and product reviews, and other health-related content, all of which support informed decision-making and continuous engagement with care. Personalized application-based technologies refer to a wide range of assistive devices that are programmed to improve care outcomes for older adults. Examples include intelligent devices that monitor vital signs such as blood pressure.

However, using ICTs to support chronic disease self-management among older adults presents significant challenges due to age-related barriers to technology use and engagement [10]. These barriers include generally lower levels of digital literacy and skills; increased concerns about privacy; and physical or cognitive limitations such as impaired vision, hearing loss, and memory decline [11-14]. As a result, older adults often depend on caregivers to effectively use health technologies [15]. In response, the literature has consistently emphasized the need for more accessible and inclusive design, particularly for ICTs aimed at this population [16]. A widely endorsed approach is to involve both older adults and their caregivers in the design process [17,18]. This co-design strategy has been shown to enhance usability, increase patient satisfaction, improve disease management, boost health literacy, and reduce health care costs [18].

Despite substantial expansion of ICTs for chronic disease management, our search found that the literature specifically focused on adults aged 65 years and older remains fragmented and inconsistent. Prior reviews often examine younger populations, focus narrowly on single diseases (eg, diabetes), or aggregate technologies without distinguishing specific self-management behaviors. To date, no comprehensive review has mapped the breadth of ICTs supporting self-management behaviors uniquely for adults aged 65 years and older.

A scoping review is methodologically suited to (1) map broad and diverse bodies of literature, (2) clarify key concepts, (3) identify types of evidence, and (4) highlight gaps rather than evaluate intervention effectiveness or pool outcomes. Given the wide variation in study designs, technologies, and outcomes and the exploratory nature of the research questions, a scoping review is justified and aligns with PRISMA-ScR (Preferred Reporting Items for Systematic Reviews and Meta-Analyses extension for Scoping Reviews) guidance.

Guided by this rationale, this scoping review aims to address the following research questions:

1. What types of ICTs have been developed to support chronic disease self-management among adults aged 65 years and older?
2. Which self-management behaviors (eg, medication adherence, physical activity, dietary management, and follow-up care) are targeted by these technologies?
3. To what extent are older adults involved in the design, adaptation, or evaluation of these technologies?

## Methods

### Protocol and Registration

This review followed the PRISMA-ScR guidelines. The protocol was not registered.

### Eligibility Criteria

Consistent with scoping review methodology, eligibility criteria were developed to capture the breadth of literature describing ICTs that support chronic disease self-management among adults aged 65 years and older, rather than to evaluate intervention effectiveness.

The inclusion and exclusion criteria are summarized in Textbox 1.

Textbox 1. **Inclusion and exclusion criteria.**

**Inclusion criteria**

- Population—adults aged 65 years or older managing 1 or more chronic diseases
- Concept—information and communication technologies (ICTs) designed to support at least 1 chronic disease self-management behavior**,** including (but not limited to) medication adherence, physical activity, diet, or follow-up appointment attendance
- Context—any setting (eg, home, community, and clinical)
- Types of sources: peer-reviewed empirical studies (qualitative, quantitative, mixed methods, feasibility or pilot studies, observational studies, or trials) published in English between 2007 and 2025

**Exclusion criteria**

- Interventions targeting only health care providers
- Nondigital or nonpersonalized technologies
- Studies not focused on chronic disease self-management
- Reviews, editorials, protocols, conference abstracts, or dissertations

A pilot screening phase was conducted during the initial article selection, in which similar articles from target journals were reviewed to refine the scope and thematic alignment of this review. This process guided database selection and search strategy refinement but was not part of the formal eligibility criteria. Because the goal of a scoping review is to map existing evidence rather than restrict it based on study design or comparator groups, no comparator was required or used as part of the eligibility criteria.

### Information Sources

A comprehensive search was conducted in 7 databases selected in collaboration with a health sciences librarian: PubMed, CINAHL, Web of Science, Cochrane Library, Compendex, IEEE Xplore, and Computers & Applied Sciences Complete. All searches were completed on December 15, 2024. Reference lists of included studies were hand searched for additional relevant articles.

### Search Strategy

Search terms were developed to reflect the review’s core aims: older adults, digital health technologies, and chronic disease self-management. Terms were adapted for each database using controlled vocabulary (eg, Medical Subject Headings [MeSH]) and keywords. Detailed search strings are included in Multimedia Appendix 1.

### Study Selection

Titles and abstracts were independently screened by 2 reviewers (PM and CRS). Rayyan, an internet-based software package, was used to facilitate article screening [19]. The 2 authors independently completed the title and abstract screening and full-text screening. Full texts of potentially eligible studies were assessed using the defined inclusion and exclusion criteria. Disagreements were resolved through consensus with the third author (YW). A PRISMA-ScR flow diagram was used to outline the study identification and selection process.

### Data Collection Process

In accordance with scoping review methodology, a standardized data charting form was developed and iteratively refined. Two reviewers (PM and CRS) independently extracted data from the included studies using a standardized data extraction form. Extracted data included study characteristics (author, year, and country), population demographics, technology description, targeted chronic diseases, self-management domain, outcomes related to technology use and acceptance, and reported effectiveness. The third reviewer (YW) resolved disagreements.

### Data Items

Key data items included the following:

- Author, year, and country
- Study design
- Participant age and health status
- Type and functionality of technology
- Chronic diseases addressed
- Self-management behaviors targeted
- Setting (home based, clinical, and other)
- Extent and type of older adult involvement in design or evaluation
- Reported outcomes (clinical, behavioral, or usability related)

Data charting was iterative; reviewers updated the form as familiarity with the literature increased, in accordance with best practices for scoping reviews.

### Synthesis of Results

Consistent with PRISMA-ScR, no risk of bias or formal quality assessment was conducted, as the goal was to describe the extent and nature of existing evidence rather than to evaluate or compare intervention effectiveness.

Because of substantial heterogeneity in study designs, outcomes, technologies, and measurement approaches, meta-analysis was neither planned nor appropriate. This aligns with the purpose of a scoping review.

### Narrative and Thematic Synthesis

A descriptive, narrative synthesis was conducted. Studies were grouped according to the following categories:

- Technology type (mobile apps, web-based platforms, wearable or sensor technologies, and smart home systems)
- Targeted self-management behaviors
- Chronic disease or health focus
- Degree of older adult involvement in design or testing

To identify key themes across studies, we used a structured thematic analysis process. First, during initial coding, 2 reviewers independently reviewed charted data and coded recurring concepts related to technology use, usability, self-management support, and design involvement. Second, in code consolidation, codes were compared, merged, and refined through discussion. Third, we used theme development to group codes into preliminary categories and iteratively refined them to generate overarching themes reflecting patterns across studies. Fourth, in a final synthesis, themes were summarized and integrated into the narrative results. This analytic process enabled identification of major trends, gaps, and characteristics of the literature.

### Effect Measures

Because this is a scoping review, no standardized effect measures were calculated. When available, quantitative outcomes (eg, percentages, use statistics, and self-reported behavior changes) were summarized descriptively based on the information reported in each study.

## Results

### Study Selection

**Figure 1.**
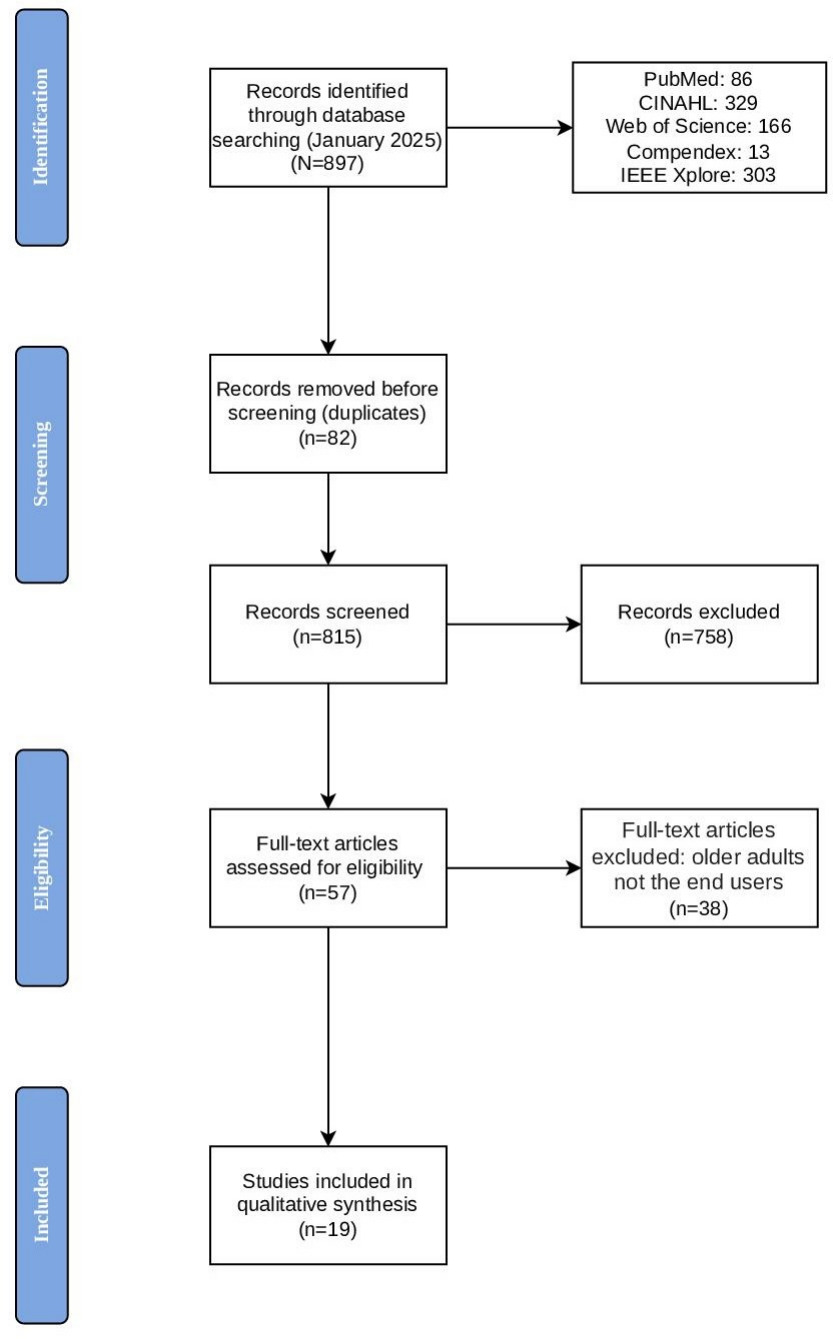
PRISMA-ScR (Preferred Reporting Items for Systematic Reviews and Meta-Analyses extension for Scoping Reviews) flow diagram illustrating the identification, screening, eligibility assessment, and inclusion of sources of evidence.

The database search identified 897 records. After deduplication, 815 (90.9%) titles and abstracts were screened. Following full-text review, 19 (2.1%) studies met the inclusion criteria. The study identification and selection process is summarized in Figure 1 (the PRISMA-ScR flow diagram).

### Characteristics of Included Studies

The 19 included studies were published between 2007 and 2025, with the majority (n=18, 94.7%) published after 2012. Studies were conducted across multiple disciplines, including rehabilitation, geriatrics, computer engineering, nursing, and digital health.

Sample sizes varied substantially, ranging from 10 to 803 participants, with most studies enrolling fewer than 120 participants. All studies included older adults aged 65 years and older, although several recruited broader adult populations and reported subgroup data for older adults.

Technologies described in the included studies (n=19) encompassed 4 primary categories: mobile apps and tablets (n=9, 47.4%), web-based platforms (n=5, 26.3%), wearable or sensor-based technologies (n=3, 15.8%), and smart home or device-integrated systems (n=2, 10.5%).

Self-management behaviors targeted most frequently included physical activity (n=11, 57.9%) and medication management (n=10, 52.6%). Dietary behavior (n=7, 36.8%) and follow-up appointment support (n=3, 15.8%) were less commonly addressed.

A detailed summary of charted study characteristics is provided in Table 1.

**Table 1.** Charted characteristics of included studies (n=19).

Study		Technology type	Self-Management focus	Age (years)	Sample size, n	Notes (major study features)
Nischelwitzer [20] 2007		Mobile medical app	Physiological tracking	36‐84	15	Early mobile prototype; pilot feasibility
Ali et al [21] 2012		Touchscreen platform	Nutritional education	60‐76	31	Education-focused dietary interface
Ammann et al [22] 2012		Web platform	Personalized physical activity	19‐89	803	Large sample; tailored content
Hess et al [23] 2012		SMS text messaging	Activity and dietary support	40‐69	47	Combined prompting system
Reeder et al [24] 2013		Digital pill dispenser	Medication management	Average 80	96	Device-integrated system
Jiménez-Fernández et al [25] 2013		Wireless sensors	Physiological tracking	Average 65	22	Wearable monitoring
Ellis et al [26] 2013		Pedometer and computer	Physical activity	Average 65.6	20	Exercise-focused
Mira et al [27] 2014		Tablet app	Medication management	≥65	99	Self-medication support
Dasgupta et al [28] 2016		Tablet app	Activity and medication management	Average 66.8	16	Multifunction platform
Costa et al [29] 2017		Smart TV system	Social and health services	≥65	62	TV-based interface
Georgsson and Staggers [30] 2017		SMS service	Follow-up, activity, and medication	Majority aged 60‐69	10	Multibehavior SMS support
Yan and Or [31] 2017		Tablet with blood pressure and glucose meters	Physiological tracking	Average 69.9	119	Integrated monitoring
Pariser et al [32] 2019		Telemedicine	Access to resources	Average 66	76	Virtual care
Lang et al [33] 2022		Tablet	Physiological tracking	≥65	116	Remote monitoring
Nambisan et al [34] 2022		Mobile app	Physical activity and diet	60‐80	20	Behavior logging
Traviss-Turner et al [35] 2024		Web program	Dietary behavior	25 to >65	22	Binge eating reduction
Shi et al [36] 2024		Web program	Exercise and diet monitoring	Average 67.2	64	Lifestyle modification
Valdeverona et al [37] 2024		SMS texting	Diet, exercise, and medications	37‐82	38	Multidomain support
Spinean et al [38] 2025		Mobile app	Physical activity and diet	18 to >65	147	Large mixed-age sample

### Mapping of Self-Management Behaviors

To describe the scope of self-management behaviors addressed by ICTs, the 19 included studies were categorized according to the primary behavior targeted.

Physical activity was addressed in 11 (57.9%) studies. These studies evaluated pedometers, activity monitoring mobile apps, wearable sensors, or web-based activity programs. Approaches included real-time step tracking, motivational prompts, and personalized exercise recommendations.

Medication management was targeted in 10 (52.6%) studies. Technologies included digital pillboxes, reminder systems, medication self-management apps, and SMS text messaging medication prompts. Most outcomes were descriptive, reporting perceived usefulness, adherence trends, or frequency of system use.

Dietary behavior was addressed in 7 (36.8%) studies. These ICTs focused on dietary logging, nutritional education through tablet or TV interfaces, and monitoring of self-reported dietary behaviors. Few studies provided objective dietary outcomes; most reported usability, satisfaction, or behavioral intentions.

Follow-up appointment support was addressed in 3 (15.8%) studies. Appointment reminders were delivered through SMS text messaging systems or integrated care platforms. Studies described improved perceived access to resources rather than clinical outcomes.

### Older Adult Involvement in Technology Design

Of the 19 studies, only 4 (21.1%) explicitly described involving older adults or caregivers in technology design or refinement. Of these, 2 studies used usability testing only, which occurred late in the development cycle. Only 2 studies incorporated participatory or co-design approaches, allowing older adults to contribute to early-stage feature development.

This limited involvement highlights a critical gap between design practices and the needs of older adult users—a key theme in our narrative synthesis.

A summary of user involvement is presented in Table 2.

**Table 2.** Extent of older adult involvement in information and communication technology design, adaptation, or evaluation (n=19).

Study	Sample size, n	Older adult involvement during design	Type of involvement
Nischelwitzer et al [20]	15	No	Initial pilot testing only
Ali et al [21]	31	No	Usability testing after development
Ammann et al [22]	803	No	Not reported
Hess et al [23]	47	No	End user feedback only
Reeder et al [24]	96	No	Postdeployment evaluation
Jiménez-Fernández et al [25]	22	No	Usability evaluation
Ellis et al [26]	20	No	Usability or feasibility testing
Mira et al [27]	99	*Yes^a^*	*Co-design: medication routine insights*
Dasgupta et al [28]	16	No	Usability and performance testing
Costa et al [29]	62	No	Informal feedback
Georgsson and Staggers [30]	10	No	Satisfaction survey
Yan and Or [31]	119	No	Logging and perceived usefulness
Pariser et al [32]	76	No	Telemedicine use feedback
Lang et al [33]	116	No	Usability testing
Nambisan et al [34]	20	*Yes*	*Iterative prototyping*
Traviss-Turner et al [35]	22	No	Behavior tracking input
Shi et al [36]	64	No	Attitudes and satisfaction assessment
Valdeverona et al [37]	38	No	Acceptability evaluation
Spinean et al [38]	147	No	End user adoption data

a Italicized text denotes studies with meaningful (but limited) co-design or development-stage input.

### Themes Identified in the Narrative Synthesis

Through thematic analysis of charted data, 3 overarching themes emerged.

#### Theme 1: ICTs Commonly Target Single Behaviors Rather Than Multidimensional Self-Management

Most technologies (14/19, 73.7%) focused on only one self-management behavior, despite the high prevalence of multimorbidity in older adults. Physical activity and medication adherence dominated the intervention landscape, while diet and follow-up behaviors were underrepresented.

#### Theme 2: Limited Integration of Older Adults in Design and Development

Consistent with prior literature on participatory design, few studies included older adults in the design process, and involvement was often superficial. Studies that incorporated older adult or caregiver feedback reported improved usability, increased engagement, and greater perceived relevance. However, participatory co-design remains the exception rather than the norm.

#### Theme 3: Focus on Usability Over Effectiveness

Across studies, outcomes overwhelmingly emphasized usability, perceived usefulness, intention to use, and satisfaction. Few studies measured changes in actual self-management behavior or clinical outcomes, reflecting a broader trend toward feasibility or proof of concept research rather than rigorous evaluation.

### Summary of Findings

The evidence base is diverse but fragmented. ICTs designed to support chronic disease self-management in older adults vary in purpose, technology type, and targeted behavior. Several key gaps were identified. These gaps included minimal involvement of older adults in technology design, scarcity of multidimensional or integrated self-management technologies, lack of objective outcome measures, and limited focus on follow-up appointment adherence and dietary behavior.

### Additional Analyses: Qualitative Synthesis

Articles that reported technology interventions and included self-management aimed at improving chronic disease outcomes using either clinical or behavioral outcomes were eligible for systematic review inclusion (Table 3). We categorized the interventions into the following 4 self-management activities: medication behavior, physical activity, dietary behavior, and follow-up appointment attendance.

**Table 3.** Self-management behaviors targeted by information and communication technologies in the included studies (n=19).

Study	Medication behavior	Follow-up appointment attendance	Physical activity	Dietary behavior	Reported outcomes related to behavior
Nischelwitzer et al [20]	No	No	Yes	No	Measured user input data including blood pressure and glucose level
Ali et al [21]	No	No	No	Yes	Measured perceived usefulness and ease of use
Ammann et al [22]	No	No	Yes	No	—^a^
Hess et al [23]	Yes	No	No	No	Measured glucose readings and appointment attendance
Reeder et al [24]	Yes	No	No	No	Measured perceived ease of use and usefulness
Jiménez-Fernández et al [25]	Yes	No	No	No	Measured degree of satisfaction and perceived ease of use
Ellis et al [26]	No	No	Yes	No	Measured walking activity and speed
Mira et al [27]	Yes	No	No	No	Measured adherence and missed doses
Dasgupta et al [28]	Yes	No	Yes	No	Measured health management skills, risk for depression, and self-reported physical activity
Costa et al [29]	Yes	Yes	Yes	No	Measured user perception
Georgsson and Staggers [30]	Yes	Yes	Yes	Yes	Measured perceived improvement
Yan and Or [31]	Yes	No	No	No	Measured actual use and perceived usefulness
Pariser et al [32]	Yes	No	No	No	Measured perceived access to clinical resources
Lang et al [33]	Yes	No	No	No	Measured actual use and perceived usefulness
Nambisan et al [34]	No	No	Yes	Yes	Measured physical activity and dietary log; condition tracking
Traviss-Turner et al [35]	No	No	No	Yes	Measured binge eating rate
Shi et al [36]	No	No	Yes	Yes	Measured improvement in exercise and dietary behaviors
Valdeverona et al [37]	Yes	—	—	—	Measured perceived usefulness and frequency of use
Spinean et al [38]	No	No	Yes	Yes	Measured adherence to physical activity recommendation and dietary recommendation

a Indicates that the applicable self-management behavior was not measured or included in the referenced study.

## Discussion

### Principal Findings

Despite the extensive body of literature on technology use in chronic disease management, relatively few studies (n=19) have explicitly examined the role of ICTs in supporting self-management behaviors among adults aged 65 years and older. This finding underscores the relative underdevelopment of an evidence base that is both age specific and behaviorally grounded, despite the high burden of multimorbidity and chronic disease management demands in this population. Given the well-established link between the 4 key self-management behaviors (ie, medication adherence, attending medical appointments, engaging in physical activity, and maintaining a healthy diet) and chronic disease outcomes, future research on technology use in chronic disease management should specifically address these behaviors to ensure more concrete and actionable findings [18].

Importantly, the scoping nature of this review allows for identification of patterns and gaps across heterogeneous study designs rather than assessment of intervention effectiveness. Viewed through this lens, the limited number of studies focused explicitly on adults aged 65 years and older reflects not only a quantitative gap but also a conceptual one in how older adults are positioned within digital health research. Future studies should place particular emphasis on adults aged 65 years and older, a population with a high prevalence of multiple chronic conditions and a documented lower intention to use health information technologies designed for self-management [39,40].

The identified gap gains further significance in light of known predictors of technology use for disease management, particularly performance expectancy and social influence. Lower levels of these factors may contribute to suboptimal adoption and sustained use of ICTs among older adults with chronic diseases [41]. While several studies implicitly acknowledged these determinants, few explicitly incorporated them into intervention design or evaluation frameworks. Understanding these predictors is crucial for informing targeted interventions that address the specific needs, expectations, and social contexts of older adults.

An increasing body of literature highlights the importance of involving older adults in the design of technologies intended to support their health [9]. However, only a small subset of studies in this review reported meaningful involvement of older adults during early design or development phases, with most limiting engagement to late-stage usability testing. Emerging research further suggests that incorporating older adults’ caregivers and support networks into the design process can improve communication, strengthen social relationships, and enhance sustained technology use [17,42]. The limited adoption of participatory and co-design approaches identified in this review therefore represents a missed opportunity to align ICT development with the lived realities of aging with chronic disease.

Regarding technology types, mobile technologies and personalized applications were the most frequently reported, surpassing web-based and home-based solutions. This pattern aligns with broader literature on technology acceptance among older adults, which suggests that tablets and smartphones are often perceived as more accessible and easier to use than desktop or laptop computers [43,44]. However, the predominance of mobile solutions should not be interpreted as evidence of optimal fit for all older adults, particularly those with sensory, cognitive, or socioeconomic barriers that may limit access or sustained use.

The included studies addressed self-management behaviors related to physical activity, medication management, diet and nutrition, and follow-up appointment attendance. While social services and social connectedness were rarely targeted, 1 study using an interactive TV-based platform demonstrated promising outcomes related to access to services and social engagement, suggesting a potential role for ICTs in addressing social isolation as a component of chronic disease self-management [45,46]. This finding highlights the need to broaden conceptualizations of self-management beyond biomedical behaviors to include social and contextual dimensions that influence health and well-being in later life.

### Implications for Practice and Future Research

This scoping review reveals several important opportunities for improving the design and implementation of ICTs that support chronic disease self-management in older adults.

First, expand the range and integration of self-management behaviors addressed. Future technologies should move beyond single-behavior interventions to reflect the multidimensional nature of chronic disease in older adults. Attention should also be given to social services as a self-management behavior, and exploration of technologies targeting social isolation is warranted.

Second, integrate older adults and caregivers across all stages of technology development. Participatory and co-design methodologies should be prioritized to ensure that ICTs align with users’ functional abilities, digital literacy, and everyday care contexts. Early-stage involvement, rather than post hoc usability testing alone, is particularly important for improving relevance and adoption.

Third, incorporate social and contextual determinants of technology use. Factors such as digital literacy, socioeconomic status, internet access, and social support networks are central to understanding ICT adoption and effectiveness among older adults. Future research should explicitly measure and report these contextual factors rather than treating them as background characteristics [43,47].

Fourth, adopt more robust evaluation approaches as technologies mature. While feasibility and usability studies remain appropriate at early stages, later-phase research should incorporate objective measures of behavior change, care processes, or health outcomes to better assess real-world impact.

Finally, develop technologies that support care continuity and follow-up. Appointment adherence and communication with health care providers remain underexplored yet highly relevant domains for older adults’ health outcomes and health care use.

Collectively, these implications underscore the need for more holistic, user-centered, and contextually informed ICT development strategies tailored to older adults managing chronic disease.

### Strengths and Limitations

This review’s strengths include a comprehensive, librarian-assisted search strategy; adherence to PRISMA-ScR reporting guidelines; and a structured, iterative approach to data charting and thematic synthesis. By focusing on self-management behaviors rather than specific diseases or technologies alone, this review offers a behaviorally grounded map of the current evidence base that complements prior disease-specific reviews.

Several limitations should be noted. First, while this scoping review provides valuable insights into ICTs targeting older adults, it did not assess intervention effectiveness or quality, consistent with scoping review methodology. Second, the heterogeneity of study designs, populations, and outcome measures limited cross-study comparability. Third, gray literature was not included, which may have excluded emerging or non–peer-reviewed technologies relevant to older adults. Finally, although sociocultural and structural factors were identified as important gaps, these were infrequently reported in the included studies, limiting deeper analysis of their influence on ICT adoption and use [46,48].

### Conclusions

This scoping review maps the current landscape of ICTs designed to support chronic disease self-management among adults aged 65 years and older, revealing a fragmented and uneven evidence base. Although a wide range of technologies has been developed, most focus on single self-management behaviors and provide limited evidence of meaningful older adult involvement in design or development.

The findings have important implications for aging researchers, health informatics scholars, and technology developers. Addressing the identified gaps, particularly the limited use of participatory design, the narrow focus on individual behaviors, and the lack of attention to social and contextual factors, will be essential for advancing more inclusive and effective ICT-based self-management support for older adults. As the population ages and the prevalence of multimorbidity increases, intentional, age-centered approaches to digital health design will be critical for improving chronic disease management and health equity in later life.

## Supplementary material

10.2196/60542 Multimedia Appendix 1 Databases and search strings.

10.2196/60542 Checklist 1 PRISMA checklist.

## Abbreviations

ICT: information and communication technology
MeSH: Medical Subject Headings
PRISMA-ScR: Preferred Reporting Items for Systematic Reviews and Meta-Analyses extension for Scoping Reviews
