# Supplementary material for: Information and Communication Technologies for Chronic Disease Self-Management in Adults Aged 65 Years and Older: Scoping Review
Source: Interact J Med Res. 2026 Mar 19;15:e60542. doi: 10.2196/60542 (PMC13002154; doi:10.2196/60542)
Supplement: Multimedia Appendix 1 [file ijmr-v15-e60542-s001.docx]

**Multimedia Appendix 1**

### Databases and strings

Our review of the seminal articles informed our selection of the following databases: PubMed, CINAHL (Cumulative Index of Nursing and Allied Health Literature), Web of Science, Cochrane, Compendex, IEEE (Institute of Electrical and Electronics Engineers), and CASC (Computers & Applied Sciences Complete). We created search strings (see Table 1) based on our themes of interest, according to specific database format. Databases included PubMed, CINAHL, Web of Science, Cochrane, Compendex, IEEE, and CASC.

Table 1. Search strings

| PubMed | CINAHL | Web of Science | Cochrane | Compen-dex | IEEE | CASC |
| --- | --- | --- | --- | --- | --- | --- |
| "Cellular Phone"[Mesh] OR "text messaging"[All Fields] OR "texting"[All Fields] OR "text message"[All Fields] OR "cell phone"[All Fields] OR "mobile phone"[All Fields] OR "mobile computing"[All Fields] OR "mhealth"[All Fields] OR "mobile health"[All Fields] OR "Tablet computer"[All Fields] OR "multimedia"[MeSH Terms] OR "multimedia"[All Fields] OR "social media"[All Fields] OR "Facebook"[All Fields] OR "Twitter"[All Fields] OR "personal health record"[All Fields] OR "personal health records"[All Fields] OR "health information technology"[All Fields] OR "health information technologies"[All Fields] OR "ehealth"[All Fields] OR “tablet”[All Fields] OR “pc”[All Fields] OR “personal health informatics”[All Fields] OR “consumer health informatics”[All Fields] | “cellular phone” or “cell phone” or “text messaging” or “text message” or texting or “mobile phone” or “mobile computing” or “mhealth” or “mobile health” or “tablet computer” or internet or blogging or “social media” or Facebook or Twitter or telemedicine or “personal health record” or “personal health records” or “computer systems” or television or “medical informatics” or “health information technology” or “health information” or “health information technology” or “ehealth” or “user-computer interface” or “computer user” or “IT Technology” or “information technology” or “tablet” or “pc” or “health informatics” or “personal health informatics” or “consumer health information” | Cellular Phone OR text messaging OR texting OR text message OR cell phone OR mobile phone OR mobile computing OR mhealth OR mobile health OR tablet computer OR telemedicine OR personal health record OR personal health records OR computer systems OR medical informatics OR health information technology OR health information technologies OR ehealth OR computer use OR user-computer interface OR computer user OR Facebook OR Twitter OR informational technology OR pc OR health informatics OR personal health informatics OR consumer health information OR consumer health informatics OR Health Care Quality, Access, and Evaluation OR medical informatics | Cellular phone or texting or text message or cell phone or mobile phone or mobile computing or mhealth or mobile health or “tablet computer” or “audio player” or “audiovisual aids” or “audiovisual” or “video” or “videos” or “multimedia” or “internet” or “blogging” or “social media” or “facebook” or “twitter” or “health records, personal” or “personal health record” or “personal health records” or “computer systems” or health or information technology or “health informatics” or “personal health informatics” or “consumer health information” or “consumer health informatics” | Cellular phone or text messaging or texting or text message or cell phone or mobile phone or mobile computing or mhealth or mobile health or tablet computer or multimedia or internet or blogging or social media or facebook or twitter or telemedicine or health records, personal or personal health record or personal health records or computer systems or medical informatics or health information technology or health information technologies or ehealth or computer user or IT Technology or information technology or tablet or pc or health informatics or personal health informatics or consumer health information or consumer health informatics | “cellular phone” or “cell phone” or “text messaging” or “text message” or texting or “mobile phone” or “mobile computing” or “mhealth” or “mobile health” or “tablet computer” or “audio player” or “audiovisual aids” or audiovisual or video or videos or multimedia or internet or blogging or “social media” or Facebook or Twitter or telemedicine or “personal health record” or “personal health records” or “computer systems” or television or “medical informatics” or “health information technology” or “health information” or “health information technology” or “ehealth” or “radio” or “reminder system” or “educational technology” or “user-computer interface” or “computer user” or “IT Technology” or “information technology” or “bluetooth” or “tablet” or “pc” or “health informatics” or “personal health informatics” or “consumer health information” or “Health Care Quality, Access, and Evaluation” | “cellular phone” or “cell phone” or “text messaging” or “text message” or texting or “mobile phone” or “mobile computing” or “mhealth” or “mobile health” or “tablet computer” or internet or blogging or “social media” or Facebook or Twitter or telemedicine or “personal health record” or “personal health records” or “computer systems” or television or “medical informatics” or “health information technology” or “health information” or “health information technology” or “ehealth” or “user-computer interface” or “computer user” or “IT Technology” or “information technology” or “tablet” or “pc” or “health informatics” or “personal health informatics” or “consumer health information” |
| “UTAUT”[All Fields] OR “usability”[All Fields] OR “patient readiness”[All Fields] OR “patient satisfaction”[All Fields] OR “patient preference”[All Fields] OR “technology acceptance model”[All Fields] OR “TAM”[All Fields] OR “ICTAM”[All Fields] OR “senior technology acceptance model”[All Fields] OR “senior technology acceptance and adoption model”[All Fields] OR “STAM”[All Fields] OR “Center for research and education on aging and technology enhancement”[All Fields] OR “CREATE”[All Fields] OR “GOMS”[All Fields] OR “GOMS model”[All Fields] OR “Goals, operators, methods, and selection rules”[All Fields] | “UTAUT” or “patient acceptance” or “usability” or “patient readiness” or “patient satisfaction” or “patient preference” or “user acceptance” or “technology acceptance” or “technology performance” or “technology influence” or “technology expectancy” or “technology facilitation” or “technology acceptance model” or “technology adoption model” or “TAM” or “ICTAM” or “senior technology acceptance model” or “senior technology acceptance and adoption model” or “STAM” or “Center for research and education on aging and technology enhancement” or “CREATE” or “GOMS” or “GOMS model” or “Goals, operators, methods, and selection rules” | UTAUT OR acceptance OR usability OR readiness OR satisfaction OR patient preference OR user acceptance OR technology acceptance OR patient compliance OR technology performance OR technology influence OR technology expectancy OR technology facilitation OR patient participation OR health care access OR technology acceptance model OR technology adoption model OR TAM OR ICTAM OR senior technology acceptance model OR senior technology acceptance and adoption model OR STAM OR Center for research and education on aging and technology enhancement OR CREATE OR GOMS OR GOMS model OR Goals, operators, methods, and selection rules | “UTAUT” or “patient acceptance” or “usability” or “readiness” or “patient preference” or “user acceptance” or “technology acceptance” or “technology expectancy” or “technology acceptance model” or “technology adoption model” or “TAM” or “ICTAM” or “senior technology acceptance model” or “senior technology acceptance and adoption model” or “STAM” or “Center for research and education on aging and technology enhancement” or “CREATE” or “GOMS model” | UTAUT or patient acceptance or usability or readiness or patient preference or user acceptance or technology acceptance or technology performance or technology influence or technology expectancy or technology facilitation or technology acceptance model or technology adoption model or TAM or ICTAM or senior technology acceptance model or senior technology acceptance and adoption model or STAM or Center for research and education on aging and technology enhancement or CREATE or GOMS or GOMS model or Goals, operators, methods, and selection rules | “UTAUT” or “patient acceptance” or “usability” or “patient readiness” or “patient satisfaction” or “patient preference” or “user acceptance” or “technology acceptance” or “technology performance” or “technology influence” or “technology expectancy” or “technology facilitation” or “technology acceptance model” or “technology adoption model” or “TAM” or “ICTAM” or “senior technology acceptance model” or “senior technology acceptance and adoption model” or “STAM” or “Center for research and education on aging and technology enhancement” or “CREATE” or “GOMS” or “GOMS model” or “Goals, operators, methods, and selection rules” | “UTAUT” or “patient acceptance” or “usability” or “patient readiness” or “patient satisfaction” or “patient preference” or “user acceptance” or “technology acceptance” or “technology performance” or “technology influence” or “technology expectancy” or “technology facilitation” or “technology acceptance model” or “technology adoption model” or “TAM” or “ICTAM” or “senior technology acceptance model” or “senior technology acceptance and adoption model” or “STAM” or “Center for research and education on aging and technology enhancement” or “CREATE” or “GOMS” or “GOMS model” or “Goals, operators, methods, and selection rules” |
| “Self care”[MeSH Terms] OR “Self care”[All Fields] OR “Health behavior”[MeSH Terms] OR “Health behavior”[All Fields] OR “patient adherence”[All Fields] OR “patient compliance”[All Fields] | “self care” or “health behavior” or “adherence” or “patient compliance” or “technology-mediated behavior” or “patient compliance” or “patient participation” or “patient satisfaction” or “patient preference” | Self care OR Health behavior OR adherence OR compliance OR technology-mediated behavior OR patient compliance OR patient participation OR patient satisfaction OR patient preference | “self care” or “health behavior” or “patient adherence” or “patient compliance” or “technology-mediated behavior” or “patient participation” or “patient satisfaction” or “patient preference” | self care or health behavior or patient adherence or patient compliance or technology-mediated behavior or patient participation or patient satisfaction or patient preference | “self care” or “health behavior” or “adherence” or “patient compliance” or “technology-mediated behavior” or “patient compliance” or “patient participation” or “patient satisfaction” or “patient preference” | “self care” or “health behavior” or “adherence” or “patient compliance” or “technology-mediated behavior” or “patient compliance” or “patient participation” or “patient satisfaction” or “patient preference” |
| “Chronic disease”[MeSH Terms] OR “Chronic disease”[All Fields] OR “recurrence”[MeSH Terms] OR “recurrence”[All Fields] OR “multiple chronic conditions”[All Fields] OR “multiple chronic conditions”[MeSH Terms] | “chronic disease” or “chronic conditions” or “mental health” or “physical health” or “recurrence” or “multiple chronic conditions” | Chronic disease OR chronic conditions OR mental health OR physical health OR recurrence OR multiple chronic conditions | “chronic disease” or “chronic conditions” or “mental health” or “physical health” or “recurrence” or “multiple chronic conditions” | chronic disease or mental health or physical health or recurrence or multiple chronic conditions | “chronic disease” or “chronic conditions” or “mental health” or “physical health” or “recurrence” or “multiple chronic conditions” | “chronic disease” or “chronic conditions” or “mental health” or “physical health” or “recurrence” or “multiple chronic conditions” |
| "Elderly"[Majr] OR "Elderly"[All Fields] OR "Older adults"[All Fields] OR "Aging population"[All Fields] OR “Elder”[All Fields] OR “Elders”[All Fields] OR "Aged"[Mesh] OR OR  “Aged”[All Fields] OR geriatric*[All Fields] OR senior*[All Fields] OR ((old[All Fields] OR oldest*[All Fields] OR older*[All Fields]) AND (person[All Fields] OR adult*[All Fields] OR individual*[All Fields] OR people[All Fields] OR patient[All Fields] OR patients[All Fields] OR inpatient*[All Fields])) | “Elderly” or “Elder” or “Older adult” or “Elders” OR aged OR geriatric* OR senior* OR ((old OR oldest* OR older) AND (person OR adult* OR individual* OR patient OR patients OR inpatient)) | Elderly OR Elders OR “Older adult” OR “Elderly population” OR aged OR geriatric* OR senior* OR ((old OR oldest* OR older) AND (person OR adult* OR individual* OR patient OR patients OR inpatient)) | Elderly or Elders or Older adults or Elder OR aged OR geriatric* OR senior* OR ((old OR oldest* OR older) AND (person OR adult* OR individual* OR patient OR patients OR inpatient)) | Elderly or Elders or Older adults or Aging population OR aged OR geriatric* OR senior* OR ((old OR oldest* OR older) AND (person OR adult* OR individual* OR patient OR patients OR inpatient)) | “Elderly” OR “Elders” OR “Old people” OR “Elderly population” OR aged OR geriatric OR senior OR ((old OR oldest OR older) AND (person OR adult OR individual OR patient OR patients OR inpatient)) | “Elderly” or “Elder” or “Older adult” or “Elders” AND “Informatics” or “Health Care Quality, Access, and Evaluation” OR aged OR geriatric* OR senior* OR ((old OR oldest* OR older) OR (person OR adult* OR individual* OR patient OR patients OR inpatient)) |
